# Triple Latency as a Driver of Chronic Inflammation: An Integrative View of HSV, EBV, and CMV Persistence in Immunocompetent Hosts

**DOI:** 10.3390/clinpract16040064

**Published:** 2026-03-24

**Authors:** Maria E. Ramos-Nino

**Affiliations:** Department of Microbiology, Immunology, and Pharmacology, St. George’s University School of Medicine, St. George P.O. Box 7, Grenada; mramosni@sgu.edu

**Keywords:** herpesviruses, HSV, EBV, CMV, triple latency, chronic inflammation, viral latency, episodic reactivation, neuroinflammation, autoimmunity, immunosenescence, biomarkers, IL-6, TNF-α, CXCL10, PGE_2_, EA-IgG, viral shedding, immunologic signatures, co-infection

## Abstract

**Background:** Herpes simplex virus (HSV), Epstein–Barr virus (EBV), and cytomegalovirus (CMV) establish lifelong latency in sensory neurons, lymphoid tissue, and myeloid–endothelial cells, respectively. A substantial proportion of adults worldwide are infected with all three viruses and may experience concurrent herpesvirus latency, yet they have largely been studied independently. This review examined whether latent and intermittently reactivating herpesviruses share overlapping inflammatory signatures and whether their combined presence contributes to chronic inflammatory burden. **Methods:** A narrative integrative review was conducted using MEDLINE, Embase, and Google Scholar (inception–October 2025). Evidence from thirty-one cohort studies and mechanistic investigations spanning virology, immunology, neurology, and clinical medicine was synthesized. **Results:** Herpesvirus reactivation rates ranged from 23% in general Intensive Care Unit (ICU) populations to 85% in severe COVID-19. Concurrent reactivation of multiple viruses occurred in 34–63% of critically ill patients and was associated with worse clinical outcomes. Notably, simultaneous CMV and EBV reactivation independently predicted mortality (adjusted hazard ratio, 3.17; 95% CI, 1.41–7.13). Across infections, overlapping inflammatory biomarkers, including IL-6, TNF-α, CRP, and PGE_2_, were consistently elevated, reflecting convergent activation of IFN and NF-κB signaling pathways. Mechanistic studies suggest cross-compartment immune priming, where CMV-driven T-cell exhaustion facilitates EBV reactivation, and viral cytokine signaling enhances HSV-associated neuroinflammation. **Conclusions:** HSV, EBV, and CMV triple latency may represent an underrecognized contributor to chronic inflammation in immunocompetent hosts. Understanding this multi-virus inflammatory network may inform mechanistic research, biomarker-guided risk stratification, and therapeutic strategies targeting convergent inflammatory pathways. Prospective interventional studies incorporating concurrent multi-virus monitoring are needed to clarify causal relationships.

## 1. Introduction

Chronic inflammation underlies a broad range of noncommunicable diseases, including cardiometabolic, autoimmune, neurodegenerative, and chronic pain disorders. Metabolic and environmental exposures are established triggers. However, by themselves, they do not account for the high degree of variability in inflammatory tone seen between individuals and across the lifespan [1].

Persistent viral infections are increasingly recognized as chronic immune regulators. By establishing lifelong latency and periodic reactivation, viruses such as herpesviruses permanently rewire the immune cell landscape, cytokine milieu, and tissue-level inflammatory responses [2,3,4,5]. Herpes simplex virus (HSV), Epstein–Barr virus (EBV), and cytomegalovirus (CMV) infect a large proportion of adults, establish latency within distinct cellular reservoirs, and undergo episodic or subclinical reactivation throughout life [6,7,8,9]. Co-reactivation of EBV and CMV has been associated with higher systemic inflammatory markers, indicating that the triple co-presence of herpesviruses is additive or synergistic in the modulation of immune homeostasis [10].

This review integrates immunologic, virologic, neurologic, and clinical evidence into a cohesive narrative in which triple latency emerges as an overlooked key driver of chronic inflammation in immunocompetent hosts.

## 2. Materials and Methods

This is a narrative integrative review aimed at synthesizing mechanistic, immunologic, neurologic, and clinical evidence for a model of triple latency (HSV, EBV, CMV) that forms the basis of chronic inflammation. Narrative review design was chosen a priori because the topic crosses virology, immunology, autoimmunity, and neurobiology, and because triple latency as a concept is nascent and has not yet coalesced in any single corpus of literature.

### 2.1. Search Strategy

PubMed/MEDLINE, Embase, and Google Scholar were searched for original research and review articles from inception through October 2025 using a structured strategy. MeSH terms were combined with free text search terms using the Boolean operators AND and OR, and relevant subject headings were mapped across databases.

Keywords were predetermined by:

(1) standardized database indexing terms for HSV, EBV, CMV, latency/reactivation; and
(2) common mechanistic “anchor terms” appearing throughout the herpesvirus literature related to immune remodeling and inflammatory signaling (e.g., IFN-I signaling, IL-6, TNF-α, CXCL10, PGE_2_, T-cell and NK-cell modulation). Biomarker terms were included to maximize capture of studies operationalizing inflammatory phenotypes and viral activity as endpoints, rather than to preferentially identify supportive findings.

Search terms included permutations of the following:

Search terms included permutations of the following concepts: (HSV-related terms), (EBV-related terms), (CMV-related terms), inflammatory mediators, and viral reactivation, combined using Boolean operators (AND/OR) as follows: (“HSV” OR “HSV-1” OR “HSV-2” OR “herpes simplex virus latency” OR “neuronal latency”) AND (“EBV” OR “Epstein–Barr virus” OR “B-cell latency” OR “LMP1” OR “LMP2A”) AND (“CMV” OR “cytomegalovirus” OR “myeloid latency” OR “immunosenescence”) AND (“chronic inflammation” OR “cytokines” OR “IL-6” OR “TNF” OR “CXCL10” OR “PGE2” OR “interferon”) AND (“viral reactivation” OR “co-reactivation” OR “latent viral burden”). Titles and abstracts were screened for relevance. Full-text review was then conducted for articles meeting the inclusion criteria. Backward and forward citation tracking of key primary studies and reviews was performed to identify additional relevant literature.

To strengthen the clinical evidence component of this narrative integrative review, a structured cohort-focused search was conducted across PubMed/MEDLINE, Embase, Google Scholar, and SciSpace (Basic Search and Full Text Search) to identify longitudinal or cohort studies evaluating combined herpesvirus exposure or reactivation (HSV, EBV, CMV) in relation to inflammatory biomarkers or inflammation-related clinical outcomes.

Database yields included SciSpace Basic Search (100 records), SciSpace Full Text Search (100 records), Google Scholar (10 records), SciSpace Library (0 records), and PubMed (2 records). After merging and deduplication, 72 unique records remained and were re-ranked by relevance to the predefined cohort question regarding associations between combined EBV, CMV, and HSV infection or reactivation and chronic inflammation.

Titles and abstracts were screened for cohort design and measurement of at least two herpesviruses, followed by full-text review of studies reporting inflammatory biomarkers (e.g., CRP, IL-6, TNF-α, immune activation markers) and/or inflammation-related clinical outcomes (e.g., mortality in critical illness, cardiovascular events, fatigue syndromes). Thirty-one distinct cohort studies met these criteria and were included in the final synthesis.

### 2.2. Inclusion Criteria

Articles were eligible for inclusion if they examined at least one of the following domains: mechanisms related to herpes simplex virus (HSV), Epstein–Barr virus (EBV), or cytomegalovirus (CMV) latency, reactivation, or immune evasion; immune or inflammatory consequences associated with latent or reactivating infection; clinical associations between viral serostatus or reactivation and chronic inflammatory disease phenotypes; evidence of synergistic or additive effects resulting from herpesvirus interaction, overlap, or co-reactivation; or mechanistic insights derived from in vitro or animal models that informed understanding of human disease processes. Studies were considered regardless of design, provided they contributed mechanistic, translational, or clinically relevant evidence aligned with the objectives of this review.

Articles were excluded if they focused exclusively on acute primary infection without describing clinically relevant latency or reactivation, did not assess immune or inflammatory endpoints, were case reports lacking mechanistic insight relevant to herpesvirus latency and immune regulation, or were non-English language publications without verified translation. Preprints were included only when peer-reviewed literature was insufficient to address a specific mechanistic point and were clearly identified as such.

### 2.3. Approach to Data Synthesis

Studies passing full-text review were synthesized iteratively in three stages:

1. Disease-specific mechanisms of latency and immune modulation for each virus
2. Identification of shared inflammatory pathways and immune remodeling across viruses (e.g., IFN-I signaling, PGE_2_ regulation, T- and NK-cell remodeling)
3. Integration of findings into a systems-level model describing how triple latency may accumulate and sustain chronic inflammatory load in immunocompetent hosts

Evidence was synthesized qualitatively across mechanistic, immunologic, and clinical domains to avoid reliance on single-study conclusions and to reduce selection bias through triangulation of independent lines of evidence.

### 2.4. Limitations

As a narrative integrative review, this study was not intended to provide an exhaustive systematic capture of all available literature. Because the primary objective was conceptual integration rather than quantitative inference, formal risk-of-bias assessment, quantitative synthesis, and meta-analysis were not performed. Methodological rigor was nonetheless strengthened through the use of multiple databases, mapped subject headings, prespecified inclusion and exclusion criteria, transparent search terminology, and iterative cross-referencing of primary studies. These measures were implemented to enhance transparency, reproducibility, and interdisciplinary coverage while remaining consistent with the goals and methodological framework of an integrative narrative review.

Cohort inference limitations. The cohort literature evaluating multiple herpesviruses simultaneously is constrained by (1) limited “true triple” designs measuring HSV, EBV, and CMV concurrently in the same participants over time; (2) heterogeneous exposure definitions (serostatus/titers vs. PCR DNAemia vs. tissue detection), which complicates cross-study synthesis; (3) confounding and reverse causality, particularly in ICU cohorts where immune dysregulation may permit reactivation (reactivation as marker) while also plausibly contributing to inflammatory injury (reactivation as driver); and (4) inconsistent measurement of inflammatory endpoints, with many community cohorts lacking paired, longitudinal cytokine/chemokine panels. Consequently, while cohort evidence supports associations between multi-herpesvirus activity and inflammation-relevant outcomes in specific contexts, it remains insufficient to establish general-population causality for triple latency without prospective multi-compartment sampling and interventional trials.

## 3. Results and Discussion

### 3.1. Latency Niches and Immune Remodeling Mechanisms of HSV, EBV, and CMV

#### 3.1.1. HSV: Neurotropic Latency and Recurrent Microinflammation

HSV-1 and HSV-2 establish lifelong latency in sensory neurons, particularly the trigeminal and sacral dorsal root ganglia, where the virus forms a reservoir capable of periodic reactivation [11,12,13], maintained by epigenetically silenced viral genomes that persist without cytolysis and allow repeated immune engagement over time [14,15,16]. In immunocompetent hosts, both genital HSV-2 and mucosal HSV-1 exhibit high rates of asymptomatic shedding, with detectable viral DNA on 5–30% of days depending on the specific virus, anatomical site, and individual host factors [17,18]. These infections are controlled locally by tissue-resident CD8^+^ T cells that suppress viral transcription while simultaneously sustaining a background inflammatory tone [19,20,21].

Each reactivation event, whether clinically overt or silent, triggers a localized burst of innate and neuroimmune mediators, including type I interferons (IFN-I), IL-6, TNF-α, prostaglandin E2 (PGE_2_), and the neuropeptide calcitonin gene-related peptide (CGRP) [22,23,24], which reinforce a persistent inflammatory tone through repeated activation of interferon-stimulated genes and Nuclear Factor Kappa B (NF-κB)–dependent pathways [23,24,25] and promote satellite glial cell activation within sensory ganglia, amplifying cytokine diffusion into surrounding neural networks [26,27,28]. Rather than resolving completely, these recurrent signaling events promote a state of immune priming characterized by sustained microglial responsiveness, altered cytokine thresholds, and amplification of subsequent inflammatory stimuli through feed-forward IFN signaling loops and persistent low-level pattern-recognition receptor (PRR) activation [29,30,31]. These cytokine and lipid signals then increase neuronal excitability, induce microglial activation, and set in motion a series of neuroinflammatory cascades that sensitize peripheral and central nociceptive pathways [32,33,34]. Cumulatively, episodic HSV reactivation functions as a chronic inflammatory driver by maintaining low-level innate immune activation and promoting long-term neuroimmune remodeling with features resembling trained innate immunity rather than classical acute resolution [35,36,37].

**Figure 1:**
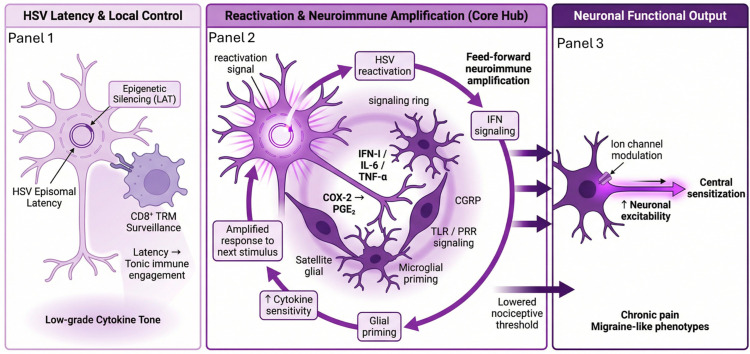
HSV latency drives a neuroimmune amplification cascade linking viral persistence to neuronal sensitization. This schematic illustrates the pathway by which latent herpes simplex virus (HSV) promotes sustained neuroimmune activation and neuronal sensitization. Panel 1 (pale lavender) depicts HSV latency within sensory neurons, characterized by episomal viral genomes, chromatin-mediated silencing of lytic genes, expression of latency-associated transcript (LAT), and non-cytolytic persistence that maintains tonic immune engagement. Panel 2 (soft violet to deep purple) highlights reactivation and neuroimmune amplification, including immune surveillance by CD8^+^ tissue-resident memory T cells (TRM), activation of pattern-recognition receptors (PRRs) such as Toll-like receptors, NF-κB–dependent signaling, and induction of inflammatory mediators including type I interferons, IL-6, TNF-α, PGE_2_, and CGRP, as well as glial priming and feed-forward interferon loops. Panel 3 (deep violet) depicts downstream neuronal outcomes, including ion channel modulation, increased excitability, central sensitization, and lowered nociceptive thresholds associated with chronic pain and migraine-like phenotypes. The progressive purple gradient (lavender to deep violet) reflects progression from viral latency to amplified neuroimmune signaling and neuronal dysfunction. Arrows indicate the direction of signaling and feed-forward amplification between neuronal, immune, and glial compartments. Upward (↑) arrows denote increases and decreases in expression or activity, respectively. Schematic generated from author-developed prompts using the FigureLabs AI platform (https://www.figurelabs.ai/, accessed on 21 March 2026) and curated by the author.

Chronic headache, migraine-like syndromes, and neuropathic pain are increasingly being linked to this inflammatory milieu (Figure 1). Epidemiologic studies show that HSV seropositivity, particularly HSV-2, is associated with a higher prevalence of severe headache and chronic pain syndromes, supporting the idea that recurrent microinflammation from HSV neurotropic latency can lead to long-term pain sensitization [38]. Mechanistically, repeated microglial activation and IFN-driven signaling have been demonstrated to sustain these processes of sensitization.

HSV latency and recurrent reactivation form a neural inflammatory axis that overlaps with EBV-driven lymphoid inflammation and CMV-driven myeloid immunosenescence to establish the broader multi-axis chronic inflammatory network that is characteristic of triple herpesvirus latency [39,40].

#### 3.1.2. EBV: B-Cell Latency, Latent Membrane Protein 1 and 2 (LMP1)/LMP2) Signaling, Autoimmunity, and Interferon Pathways

EBV establishes lifelong latency within memory B cells through a restricted viral gene program optimized for immune evasion, cell survival, and periodic reactivation, reinforced by epigenetic remodeling of host chromatin and viral episomes that maintain a metabolically active yet immunologically persistent state [41,42,43]. The two latent membrane proteins LMP1 and LMP2A are particularly important for these activities. LMP1 mimics CD40, functioning as a constitutively active signaling complex that activates canonical and non-canonical NF-κB, Janus kinase/Signal transducer and activator of transcription (JAK/STAT), and Activator protein 1 (AP-1) pathways, thereby promoting B-cell survival, proliferation, metabolic fitness, and resistance to apoptosis. LMP1-driven NF-κB and JAK/STAT signaling enhances metabolic fitness by increasing glycolytic flux, mitochondrial biogenesis, and lipid biosynthesis, thereby sustaining the proliferation of infected B cells. Concurrently, LMP1 upregulates anti-apoptotic proteins, including BCL-2, BCL-XL, and MCL-1, while suppressing pro-apoptotic signaling by inhibiting caspase activation and modulating the p53 pathway, allowing EBV-infected B cells to evade programmed cell death and persist long term within the memory B-cell compartment [44,45]. In addition, LMP1 induces expression of inflammatory cytokines such as IL-6, B cell activator factor (BAFF), and TNF-family ligands, thereby reshaping the surrounding lymphoid microenvironment and reinforcing pro-survival signaling circuits [41,44]. LMP2A further supports EBV persistence by providing a tonic, antigen-independent B-cell receptor (BCR)–like signal through recruitment of Src-family kinases, including Lyn and Syk, and activation of downstream phosphatidylinositol 3-kinase (PI3K)/protein kinase B (Akt) signaling. This signaling enables infected B cells to bypass normal BCR survival checkpoints while maintaining basal PI3K/Akt activity that lowers activation thresholds and favors chronic immune activation [45,46].

EBV’s reprogramming of the B-cell compartment strongly predisposes to autoimmunity. A defining feature is the expansion of T-bet^+^ B cells. This pro-inflammatory B-cell subset develops under IFN-γ–driven inflammatory conditions and in response to Toll-like receptor (TLR) stimulation, which act as potent antigen-presenting cells (APCs) and producers of pathogenic IgG. Recent data demonstrates that EBV can convert autoreactive B cells into hyperactive APCs, directly linking EBV infection to aberrant antigen presentation and spontaneous germinal center activity that are hallmarks of systemic lupus erythematosus (SLE) [47,48] through sustained upregulation of costimulatory molecules (CD80/CD86) and enhanced MHC class II presentation [45,47]. These T-bet^+^ cells drive epitope spreading, the progressive diversification of immune responses from the initial antigenic epitope to additional epitopes on the same or distinct self-antigens, chronic germinal center activation, and sustained autoimmune responses by maintaining prolonged interactions with T follicular helper cells (Tfh) and promoting IFN-γ–skewed immune circuits [48,49].

Molecular mimicry further potentiates autoimmune risk. EBV proteins, especially EBNA1, share structural similarity with host nuclear antigens, allowing the formation of cross-reactive autoantibodies and diversification of autoreactive B-cell repertoires [42,50]. Meanwhile, persistent EBNA-driven transcriptional programs alter DNA-damage responses and oxidative stress pathways, reinforcing inflammatory signaling [45,50]. This mimicry underlies the wide range of autoantibodies seen in SLE and contributes to disease propagation via polyreactive B-cell activation.

**Figure 2:**
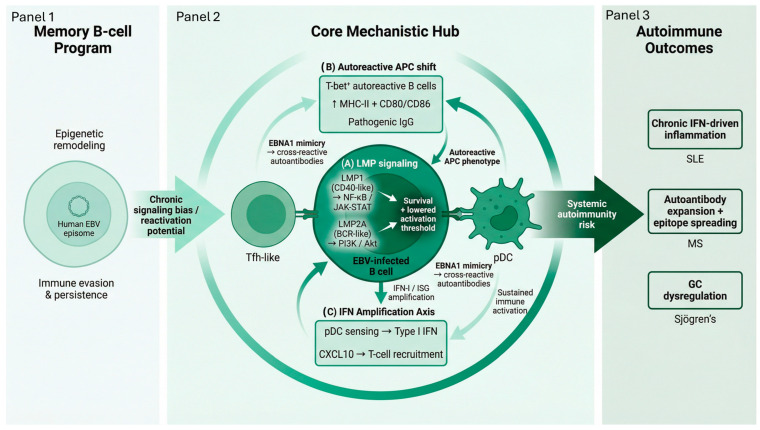
EBV latency promotes autoreactive B-cell programming and type I interferon–driven autoimmune amplification. This schematic illustrates the pathway linking latent Epstein–Barr virus (EBV) infection to systemic autoimmune activation. Panel 1 (pale mint) depicts EBV persistence within memory B cells, characterized by episomal viral genomes, epigenetic remodeling, and immune evasion that sustain long-term latency and chronic B-cell signaling. Panel 2 (emerald green) represents the central reprogramming hub, where EBV latent membrane proteins LMP1 and LMP2A mimic host signaling to activate NF-κB/JAK-STAT and PI3K/Akt pathways, promoting survival of T-bet^+^ autoreactive B cells and enhanced antigen presentation (↑ MHC-II, CD80/CD86). Molecular mimicry mediated by EBNA1 and type I interferon/CXCL10 signaling further amplifies immune activation through interactions with T follicular helper cells (Tfh) and plasmacytoid dendritic cells. Panel 3 (deep forest green) illustrates downstream systemic outcomes, including chronic interferon-driven inflammation, dysregulated germinal center responses, expansion of autoantibody-producing cells, and increased risk for autoimmune diseases such as systemic lupus erythematosus, multiple sclerosis, and Sjögren’s syndrome. The left-to-right green gradient (pale mint to deep forest) represents progression from latent viral persistence to immune reprogramming and autoimmune amplification. Arrows indicate the direction of signaling, progression, and feedback interactions within the EBV-driven network. Upward (↑) arrows denote increased expression or activity. Schematic generated from author-developed prompts using the FigureLabs AI platform (https://www.figurelabs.ai/, accessed on 21 March 2026) and curated by the author.

EBV reactivation leads to heightened type I IFN (IFN-I) signaling and production of CXCL10, creating a self-perpetuating inflammatory circuit that recruits activated T cells and drives persistent immune activation [42,51] through plasmacytoid dendritic cell sensing of viral nucleic acids and amplification of interferon-stimulated gene networks [52,53]. Chronic exposure to these IFN-rich environments promotes immune exhaustion, dysregulated germinal center architecture, and sustained cytokine gradients that extend beyond lymphoid tissue into systemic circulation [48,51]. Together, these mechanisms establish EBV as a major viral amplifier of autoimmune disease. Cumulative mechanistic and epidemiologic data now implicate EBV in the etiology or progression of SLE, multiple sclerosis, and Sjögren’s syndrome [42,47,51] (Figure 2).

#### 3.1.3. CMV: Myeloid Latency, Immunosenescence, and NK-Cell Evasion Mechanisms

CMV establishes lifelong latency primarily in monocytes, dendritic cells, and endothelial cells, forming a persistent reservoir that continuously influences host immunity through chronic antigenic stimulation and periodic reactivation [9,54]. Latent infection is maintained during myeloid differentiation, with viral gene expression kept at low levels yet sufficient to imprint durable transcriptional and epigenetic changes on infected cells, thereby biasing them toward a pro-inflammatory phenotype [54,55,56]. A hallmark of CMV-mediated immune remodeling is the expansion of senescent CD8^+^CD28^−^ T cells, a population of T cells characterized by high cytotoxic potential, limited proliferative capacity, and a pro-inflammatory secretory profile. The accumulation of these cells promotes immunosenescence and drives inflammaging, even in otherwise healthy adults [57,58], by skewing the T-cell repertoire toward oligoclonal “memory inflation,” defined as the progressive expansion and long-term persistence of antigen-experienced T cells during chronic viral antigen exposure. This process reduces naïve T-cell pools and **sustains** tonic cytokine production (e.g., IFN-γ, TNF-α) that perpetuates low-grade systemic inflammation [57,58].

Equally important, and often underrecognized, are CMV’s NK-cell evasion and modulation strategies, which have profound effects on innate immune surveillance. To evade CD8^+^ T cells, CMV downregulates MHC class I molecules using viral proteins such as US2, US3, US6, and US11 through disruption of antigen presentation pathways, including MHC-I retention/degradation and impaired peptide loading in the endoplasmic reticulum [9,59]. It then counterintuitively expresses MHC-I decoy molecules such as UL18, which binds to an inhibitory NK-cell receptor LIR-1, thereby suppressing NK-mediated cytotoxicity [60,61] and shifting NK responses toward inhibitory signaling dominance, allowing infected cells to persist despite “missing-self” cues [60].

CMV additionally manipulates ligands for the activating NK-cell receptor Natural killer group 2, member D (NKG2D), a critical sensor of cellular stress and infection. Viral proteins such as UL16 and UL142 retain or downregulate NKG2D ligands like MICB, ULBP1, and ULBP2, thus reducing NK-cell activation and facilitating viral persistence [62,63,64]. In parallel, CMV shapes the NK compartment by driving expansion of adaptive-like NKG2C^+^ NK cells with altered receptor repertoires and long-lived, epigenetically imprinted effector programs, which further remodel innate immune tone over time [65,66].

**Figure 3:**
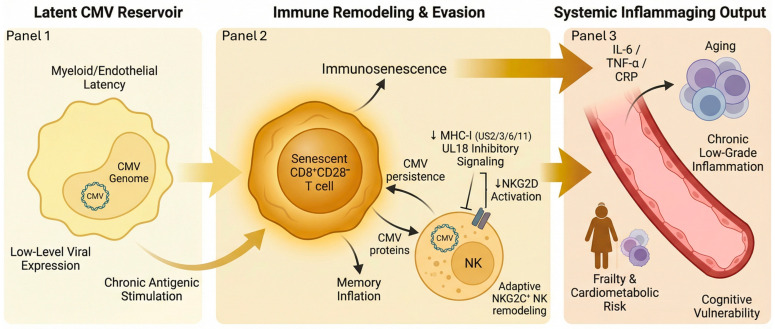
CMV latency drives immunosenescence and systemic inflammaging through sustained immune remodeling. This schematic illustrates the pathway linking latent cytomegalovirus (CMV) infection to immune aging and chronic inflammatory outcomes. Panel 1 (pale yellow) depicts CMV latency within myeloid and endothelial reservoirs, characterized by episomal persistence, low-level viral gene expression, and continuous antigenic stimulation that primes long-term immune engagement. Panel 2 (mustard) represents the central immune-remodeling hub, where persistent CMV exposure promotes expansion of senescent CD8^+^CD28^−^ T cells (“memory inflation”) and functional immunosenescence. Viral immune-evasion mechanisms—including MHC-I downregulation (US2/3/6/11), UL18-mediated inhibitory signaling, and reduced NKG2D activation—modulate NK-cell responses and promote expansion of adaptive NKG2C^+^ NK cells. Together, these processes sustain chronic immune activation while limiting effective viral clearance. Panel 3 (deep amber) illustrates downstream systemic consequences, including elevated inflammatory mediators (IL-6, TNF-α, CRP), chronic low-grade inflammation, and increased risk of frailty, cardiometabolic dysfunction, and cognitive vulnerability. The left-to-right color gradient (pale yellow to deep amber) reflects progression from latent viral persistence to immune remodeling and inflammaging phenotypes. Arrows indicate the direction of progression and feedback interactions within the CMV-driven pathway. Downward (↓) arrows denote increases and decreases in expression or activity, respectively. Schematic generated from author-developed prompts using the FigureLabs AI platform (https://www.figurelabs.ai/, accessed on 21 March 2026) and curated by the author.

These combined immune evasion programs allow CMV-infected myeloid cells to persist while continually perturbing host immune equilibrium, including sustained monocyte activation, endothelial inflammatory signaling, and increased baseline expression of inflammatory mediators that promote vascular dysfunction and tissue-level inflammaging [55,56,67]. Persistent CMV seropositivity is strongly associated with higher IL-6, TNF-α, and CRP, greater frailty, and adverse cardiometabolic and cognitive outcomes in aging adults [68,69,70] (Figure 3), consistent with CMV acting as a chronic antigenic driver that reinforces systemic cytokine tone and accelerates age-associated immune remodeling [57,58]. Through sustained modulation of NK-cell and T-cell surveillance, CMV exerts a chronic, system-wide reshaping of innate and adaptive immunity that potentiates the inflammatory burden imposed by HSV and EBV, amplifying the multi-axis inflammatory network characteristic of triple latency by coupling myeloid inflammaging to lymphoid and neuroimmune axes, thereby lowering the threshold for persistent inflammatory activation across tissues [9,56].

### 3.2. Synergy of Triple Latency: A Systems-Level Inflammatory Model

#### 3.2.1. Multi-Compartment Inflammatory Load

HSV, EBV, and CMV establish lifelong latency within distinct anatomical and cellular niches, sensory neurons for HSV [11,14], memory B cells for EBV [41,43], and monocyte-lineage myeloid and endothelial cells for CMV [9,54]. This spatial compartmentalization creates a distributed “inflammatory architecture” in which each reservoir generates unique immune signals at tissue interfaces and within the circulation.

Because each virus occupies a distinct immune niche, their coexistence produces a multi-compartment inflammatory load that exceeds the effects of individual infections [71,72,73,74]. Population-based studies support this cumulative-burden model, showing that combined herpesvirus exposure correlates with elevated IL-6 and CRP levels and immune remodeling in aging cohorts [75,76,77,78,79,80,81]. Intensive care unit (ICU) cohorts further demonstrate that simultaneous herpesvirus reactivation identifies a high-risk inflammatory phenotype associated with worse clinical outcomes. In one critically ill adult cohort, CMV and Human herpesvirus 6 (HHV-6) coreactivation was associated with a significantly higher composite endpoint of death or continued hospitalization at day 30 (76% vs. 41% in patients without viral reactivation; adjusted OR 7.5, 95% CI 1.9–29.9) [82], supporting a clinically relevant multi-virus effect rather than isolated viral activity. Similar patterns of viral reactivation have been observed in other ICU populations. In septic shock patients, herpesvirus viremia was detected in 68% of cases, with multiple concurrent viral reactivations occurring in approximately 34% of patients; EBV was the most frequently detected virus (48%), and concurrent CMV and EBV viremia was independently associated with increased mortality (adjusted subdistribution hazard ratio (SHR) 3.17, 95% CI 1.41–7.13) [83]. In critically ill COVID-19 patients, herpesvirus reactivation was observed in 85% of cases, with EBV detected in 65% of patients and multiple viral reactivations occurring in 63% of the cohort. Importantly, viral reactivation was associated with increased mortality risk (OR 2.46, 95% CI 1.02–5.89), highlighting the potential clinical relevance of multi-virus reactivation during severe inflammatory states [84].

#### 3.2.2. Crosstalk Mechanisms

Latent herpesviruses reinforce systemic immune perturbation through cross-compartment signaling. Chronic CMV infection drives T-cell exhaustion and immune senescence [57,58,68], weakening cytotoxic surveillance and permitting EBV reactivation and B-cell expansion [74,85]. EBV-associated cytokines, including type I interferons and CXCL10 [52,53], promote autoimmune signaling while sensitizing neural tissues, increasing susceptibility to HSV-associated neuroinflammation [72,86]. Human immunophenotyping cohorts demonstrate that CMV/EBV coinfection expands effector-memory T-cell pools and produces aging-like immune profiles [87], suggesting that viral crosstalk reshapes baseline immune tone rather than acting as episodic events. Conversely, HSV-driven microglial priming amplifies systemic inflammatory cascades, enhancing antigen presentation and CMV-mediated myeloid activation [55,69]. These bidirectional interactions generate a self-reinforcing feedback network that magnifies composite inflammatory load [72,86], consistent with cohort observations linking multi-herpesvirus reactivation to heightened inflammatory signaling during severe illness [82,88].

#### 3.2.3. Prostaglandin Amplification

Prostaglandin E_2_ (PGE_2_), generated through the cyclooxygenase-2 (COX-2) pathway, represents a convergent inflammatory amplifier during herpesvirus latency. HSV, EBV, and CMV each induce COX-2 expression and PGE_2_ release during reactivation or persistent infection [89,90,91,92], amplifying cytokine signaling and immune cell recruitment. Elevated PGE_2_ sensitizes nociceptors and promotes neuroinflammation [93,94,95,96], mechanisms implicated in chronic pain syndromes and trigeminal autonomic cephalalgia-like (TAC-like) headaches [38,97,98].

Observational studies linking herpesvirus seropositivity with chronic pain and inflammatory biomarkers [38,99,100,101] provide indirect human support for this prostaglandin-driven amplification axis. This pathway, therefore, offers a mechanistic bridge between viral latency, neuroinflammatory pain, and headache susceptibility [102,103,104].

#### 3.2.4. Microglial Priming and Neuroinflammation

Triple herpesvirus latency promotes sustained microglial priming and neuroimmune activation. Repeated exposure to HSV reactivation signals and EBV/CMV-driven cytokine surges primes microglia to respond more vigorously to secondary insults [19,23,44,70,105,106,107], amplifying pro-inflammatory mediator release and lowering thresholds of nociceptive and cognitive pathways [108,109].

**Figure 4:**
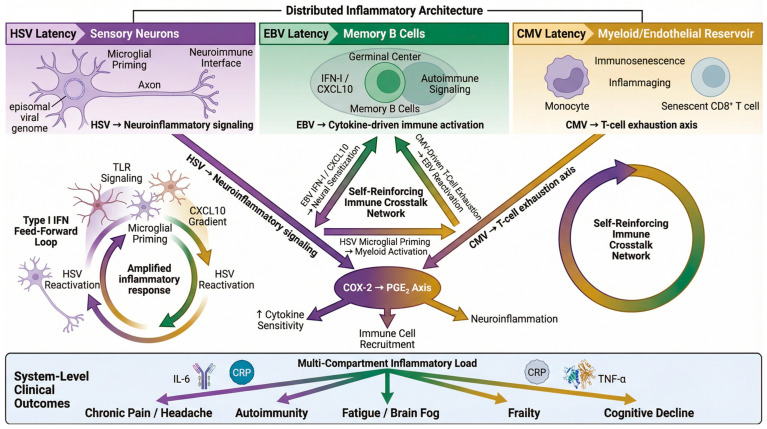
Systems-level model of multi-compartment inflammatory signaling during triple herpesvirus latency. This schematic illustrates a proposed conceptual framework derived from convergent mechanistic and clinical evidence showing how concurrent latency of herpes simplex virus (HSV), Epstein–Barr virus (EBV), and cytomegalovirus (CMV) could generate a distributed inflammatory architecture across neuroimmune, lymphoid, and myeloid compartments. Because few studies have directly measured triple latency in the same individuals, this model should be interpreted as a hypothetical integrative scenario rather than a proven biological sequence. HSV (purple), EBV (green), and CMV (mustard) persist in distinct latency niches—sensory neurons, memory B cells, and myeloid/endothelial cells—where episodic or subclinical reactivation produces intermittent immune signaling. HSV latency promotes microglial priming and neuroinflammatory signaling; EBV latency drives interferon/CXCL10-mediated lymphoid activation and autoimmune skewing; and CMV persistence induces T-cell exhaustion and immunosenescence. Cross-compartment crosstalk may reinforce inflammatory signaling, including CMV-driven immune aging, facilitating EBV reactivation, EBV-derived cytokines sensitizing neural pathways, and HSV-associated neuroimmune activation, enhancing systemic antigen presentation. Convergent activation of the COX-2/PGE_2_ axis may further amplify cytokine signaling and neuroinflammation, linking spatially distributed viral reservoirs to shared outcomes such as chronic pain, fatigue, autoimmunity, frailty, and cognitive vulnerability. Potential counter-regulatory or inhibitory interactions between these pathways may also occur; however, they remain insufficiently characterized. The model illustrates how distinct latency niches may collectively contribute to systemic inflammatory load even in the absence of overt viral symptoms. Arrows indicate the direction of signaling, cross-compartment interactions, and feed-forward amplification within the distributed inflammatory network. Circular arrows represent self-reinforcing feedback loops. Upward (↑) arrows denote increased expression, activity, or sensitivity. Schematic generated from author-developed prompts using the FigureLabs AI platform (https://www.figurelabs.ai/, accessed on 21 March 2026) and curated by the author.

Emerging cohort data suggest that individuals with multiple latent herpesvirus exposures demonstrate increased neuroimmune vulnerability, including fatigue, cognitive slowing, and inflammatory biomarker elevation [110,111,112,113,114,115,116]. Together, these findings support a model in which distributed viral latency drives chronic neuroimmune activation associated with pain sensitization, “brain fog,” and persistent fatigue, even in the absence of overt viral symptoms (Figure 4).

#### 3.2.5. Cohort-Level Evidence for Multi-Herpesvirus Exposure/Reactivation

A synthesis of 31 cohort studies (29 prospective/longitudinal and 2 retrospective) contextualizes this systems-level model. Although definitive causal chains remain unproven, several consistent patterns emerge, summarized in Table 1.

As shown in Table 1, the clinical literature is strongest for CMV-associated immunosenescence and inflammaging, EBV-associated autoimmune amplification (including conversion of autoreactive B cells into hyperactive antigen-presenting cells), and HSV-associated neuroinflammatory pain phenotypes. Dual-virus and multi-herpesvirus studies, particularly in aging and critical illness cohorts, further support the idea that combined herpesvirus burden is associated with greater immune dysregulation than single-virus exposure alone. For example, the Bennett et al. study [124] demonstrated that EBV and CMV co-reactivation in older adults correlated with elevated systemic inflammatory markers, while ICU studies [82,83,84,125] showed that multi-herpesvirus reactivation identifies high-risk inflammatory phenotypes with increased mortality. However, because few studies concurrently measure HSV, EBV, and CMV longitudinally in the same participants, the systems-level concept of triple latency should be interpreted as an integrative framework grounded in convergent but still incomplete clinical evidence. Notably, some studies (e.g., [128]) reported null findings for associations between CMV antibody levels and cardiovascular outcomes, underscoring the need for standardized measurement approaches and prospective multi-virus cohort designs.

**Critical illness cohorts**: In critically ill populations, herpesvirus reactivation is common and has been associated with disease severity and mortality. Multiple herpesvirus reactivation has been documented in septic shock cohorts. In a prospective ICU study of previously immunocompetent patients with septic shock, herpesvirus viremia was detected in 68% of patients, with individual prevalence rates of 48% for EBV, 26% for HSV-1, 24% for HHV-6, and 18% for CMV [123]. Importantly, multiple concurrent viral reactivations occurred in 34% of patients, and concurrent CMV and EBV viremia was independently associated with increased mortality (adjusted subdistribution hazard ratio 3.17, 95% CI 1.41–7.13) [83]. In mechanically ventilated patients with severe pneumonia, viral reactivation in the lungs has been associated with increased mortality [125]. In critically ill COVID-19 patients with severe pneumonia, reactivation of EBV, CMV, and HSV has been documented. In one ICU cohort, viral reactivation occurred in 85% of patients, with multiple herpesvirus reactivations observed in 63%, and EBV being the most frequently detected virus (65%). Viral reactivation was independently associated with increased in-hospital mortality (OR 2.46, 95% CI 1.02–5.89) as well as higher rates of ventilator-associated pneumonia and bloodstream infection [84].

Studies evaluating simultaneous herpesvirus reactivation further demonstrate the clinical significance of multi-virus activity. For example, in a cohort of critically ill adults, HHV-6 viremia occurred in 23% of patients, and most patients with HHV-6 reactivation also reactivated CMV (70%) [82]. Importantly, the composite endpoint of death or continued hospitalization at day 30 occurred in 76% of patients with HHV-6 and CMV coreactivation compared with 41% of those without viral reactivation, and co-reactivation remained independently associated with worse outcome in multivariable analysis (adjusted OR 7.5, 95% CI 1.9–29.9) [82].

Although some ICU cohorts include HHV-6 alongside CMV, EBV, and HSV, the present review focuses on the triple-latency framework involving HSV, EBV, and CMV, which are the most prevalent persistent herpesviruses in the general population and for which mechanistic data are most robust. These observations nonetheless illustrate a broader phenomenon in which simultaneous herpesvirus reactivation identifies a high-risk inflammatory phenotype during severe illness, supporting the concept that cumulative viral activity may amplify systemic immune dysregulation (Table 2).

**Community and aging cohorts:** Evidence is heterogeneous and often based on serostatus rather than active reactivation measures. CMV seropositivity and higher pathogen burden are associated with mortality, frailty, and inflammatory markers in several cohorts [117,118,119]. However, null findings exist when only antibody titers are analyzed [128].

**Cardiovascular cohorts**: Associations are more consistent for CMV than HSV or EBV, linking higher antibody levels to coronary disease risk and subclinical atherosclerosis in some populations [120,121,122]. Meanwhile, concurrent measurement of all three viruses remains uncommon.

**Pediatric cohorts**: Early-life CMV/EBV coinfection shapes effector-memory T-cell architecture without impairing vaccine responses, suggesting that herpesvirus persistence establishes long-term immune tone [127].

In synthesis, multi-herpesvirus reactivation robustly marks inflammatory dysregulation in critical illness, whereas community-level evidence supports a cumulative pathogen-burden model dominated by CMV-linked immune remodeling. These data align with a distributed, multi-compartment framework of herpesvirus-driven inflammation, while highlighting gaps in temporal, measurement, and causal analyses.

### 3.3. Clinical Implications

Triple latency with HSV, EBV, and CMV has extensive clinical implications, as the combined effects of viral persistence and episodic reactivation can underline chronic, multisystem inflammatory phenotypes even in immunocompetent individuals. Accumulating evidence links these viruses, individually and in some contexts jointly, to chronic daily headache and TAC-like syndromes [38,129], autoimmune activation and autoantibody expansion [123,130], fatigue and post-infectious neuroimmune syndromes [110,131,132], low-grade metabolic inflammation and insulin resistance [124,133], frailty and reduced physiological reserve in aging adults [126,134], and progressive cognitive decline and neuropsychiatric vulnerability [135,136]. Recent cohort studies have found that co-reactivation of EBV and CMV is associated with higher systemic inflammatory markers and accelerated immunosenescence, supporting a multiplicative rather than additive effect of triple latency on long-term health [124,137].

A diverse panel of immune, inflammatory, and virologic biomarkers may serve as candidate indices of latent viral activity in future prospective studies. Elevated cytokines (IL-6, TNF-α), chemokines (CXCL10), C-reactive protein (CRP), and prostaglandin mediators (PGE_2_) reflect downstream inflammatory activity [138,139]. Virologic indicators, such as EBV early antigen (EA)-IgG, CMV IgG titers, HSV shedding frequency, and multiplex PCR detection of subclinical viral reactivation, provide additional and complementary insights into the intensity of latent viral activity [110,124,138]. Collectively, these biomarkers offer a framework for quantifying the systemic and compartment-specific inflammatory load associated with triple latency and can serve as candidates for future risk stratification, mechanistic studies, and interventional trials.

### 3.4. Limitations of Current Evidence, Research Gaps, and Future Directions

Several limitations of the current evidence base should be acknowledged. Many studies summarized in this review rely on serological markers or viral DNA detection, which may reflect prior exposure or intermittent viral shedding rather than definitive evidence of active viral reactivation. In addition, few studies simultaneously measure HSV, EBV, and CMV within the same cohort, limiting rigorous evaluations of true multi-virus or “triple-latency” interactions. Consequently, much of the available literature demonstrates associations between viral markers and inflammatory or clinical outcomes rather than clear causal relationships. Differences in cohort characteristics, sampling timing, and detection methods further complicate cross-study comparisons and interpretation.

Beyond these methodological limitations, major gaps remain in our understanding of the cumulative effects of HSV, EBV, and CMV latency on chronic inflammation. There is a relative dearth of longitudinal, systems-level studies that integrate virome, immunome, and tissue-specific data across the lifespan, and few studies systematically track multi-virus reactivation using serial, multi-compartment sampling. The references cited in this review reflect most key themes in recent high-quality primary and review literature. However, as with most syntheses in this rapidly evolving field, the reference list may not provide exhaustive coverage of the latest developments in single-cell, spatial, or multi-omics analysis of tissue-specific latency, nor of recent advances in neuroimaging and high-throughput systems immunology. These areas are under active investigation, and newly published studies continue to expand our understanding of herpesvirus latency and its clinical consequences [77,86]. Likewise, neuroimaging studies synchronized with virological and immunological measures are largely absent, leaving connections between subclinical viral activity, neuroinflammation, and clinical outcomes insufficiently defined.

Future work should focus on prospective, multi-compartment cohort designs that incorporate serial sampling and multi-omics platforms to map viral latency, reactivation dynamics, and immune remodeling. Broader and standardized biomarker panels, including cytokines, chemokines, viral antigens, and microRNAs, should be integrated into harmonized biobank and assay frameworks to enable cross-study comparisons [140].

Advances in neuroimaging [Positron Emission Tomography (PET), functional Magnetic Resonance Imaging (fMRI), MR spectroscopy] should be paired with virome and immunologic profiling to delineate CNS effects of intermittent viral activity. Population-level registries, digital health tools, and biosensing technologies may help capture subclinical reactivation patterns in immunocompetent hosts, while animal models can elucidate synergistic mechanisms underlying triple latency [141].

Finally, consortium-based data-sharing initiatives and globally representative cohorts are essential to support reproducibility, improve mechanistic understanding, and accelerate translational efforts, including trials targeting convergent inflammatory pathways like COX-2/PGE_2_ or microglial priming. These strategies collectively aim to close the core knowledge gaps surrounding the true impact of triple herpesvirus latency on chronic inflammation and disease [142].

## 4. Conclusions

Triple latency with HSV, EBV, and CMV establishes a distributed, life-long inflammatory architecture spanning neuronal, lymphoid, and myeloid–endothelial compartments. Even in immunocompetent hosts, the sum of episodic HSV neuroinflammation, EBV-driven lymphoid activation and autoimmunity, and CMV-associated immunosenescence may contribute to a persistent, multi-axis inflammatory load that can influence systemic immune tone across the lifespan. This review spotlights triple latency as an underrecognized and pervasive contributor to chronic inflammatory phenotypes, including chronic daily headache, autoimmune priming, fatigue syndromes, frailty, and neurocognitive decline.

Recognizing herpesvirus persistence as a hidden, multi-compartment architecture of chronic inflammation provides a conceptual foundation for identifying shared biomarkers and convergent inflammatory pathways. Integration of virologic, immunologic, and neuroimmune perspectives may help with the development of targeted interventions that modulate virus-driven inflammation, attenuate microglial sensitization, and ultimately mitigate disease burden. A systems-level framework for triple latency can therefore guide future mechanistic research, biomarker discovery, and translational strategies aimed at limiting the cumulative impact of latent herpesviruses on long-term health.

## Figures and Tables

**Table 1:** Representative clinical evidence for single-, dual-, and multi-herpesvirus exposure/reactivation in relation to inflammatory and clinical outcomes.

Exposure Category	Study/Context	Viral Measure(s)	Main Inflammatory/Immune Findings	Clinical Association/Outcome	Key Interpretation
Single latency: CMV	Older adult/community cohorts [117,118,119]	CMV seropositivity or antibody levels	Higher IL-6, TNF-α, CRP; immune-risk phenotype; T-cell aging signatures	Frailty, all-cause mortality, cardiovascular mortality, cognitive vulnerability	CMV has the strongest cohort-level association with immunosenescence/inflammaging outcomes
Single latency: CMV	Cardiovascular cohorts [120,121,122]	CMV serology/antibody levels	Pro-inflammatory vascular milieu; indirect evidence of endothelial/immune activation	Coronary disease risk, carotid intima-media thickening, subclinical atherosclerosis	CMV is more consistently linked than HSV or EBV to vascular-inflammatory outcomes
Single latency: HSV	Adult population cohort/cross-sectional headache study [38]	HSV-2 seropositivity	Indirect support for inflammatory pain signaling	Higher prevalence of severe headache/migraine-like syndromes (aged 20–49 years)	HSV-associated latency/reactivation may contribute to chronic neuroinflammatory pain phenotypes
Single latency: EBV	Autoimmune/mechanistic-clinical linkage studies [47,51,123]	EBV serostatus/reactivation markers; disease-linked immune signatures	Type I IFN/CXCL10 signaling, autoreactive B-cell programming (T-bet^+^ B cells), APC activation	SLE, MS, Sjögren’s syndrome, autoimmune amplification	EBV is strongly linked to autoimmune-skewed inflammatory biology; EBV converts autoreactive B cells into hyperactive APCs
Dual latency/reactivation: EBV + CMV	Aging/immune-phenotyping cohorts [87]	EBV and CMV coinfection/serostatus	Expanded effector-memory T-cell pools; aging-like immune profile	Immune remodeling without overt acute disease	Dual herpesvirus exposure can reshape baseline immune tone beyond single-virus effects
Dual reactivation: EBV + CMV	Older adults/stress-inflammation cohort [124]	EBV and CMV reactivation/antibody indices	Higher systemic inflammatory markers (IL-6, TNF-α)	Greater inflammatory burden in aging adults	Reactivation burden, not just seropositivity, may track chronic inflammation more closely
Dual reactivation: HHV-6 + CMV	ICU/critical illness cohort [82]	Viral reactivation by PCR (plasma DNAemia)	High inflammatory-risk phenotype	Composite endpoint of death or continued hospitalization at day 30 was higher with HHV-6/CMV coreactivation (76% vs. 41% without viral reactivation)	Simultaneous herpesvirus reactivation marks severe inflammatory dysregulation in acute illness
Multi-herpesvirus reactivation	Septic shock/ICU cohorts [83,84,125]	Multiple herpesvirus viremia/reactivation (including CMV, EBV, HSV in some cohorts)	Broad immune activation; inflammatory dysregulation	Increased mortality, worse ICU outcomes, severe pneumonia/COVID-19 complications; EBV most prevalent (48%), and CMV–EBV co-reactivation independently predicted mortality (adjusted SHR 3.17)	Multi-virus reactivation is clinically relevant in critical illness, though causality remains difficult to infer
Pathogen burden/multiple chronic infections	Community and elderly cohorts [76,79,118,126]	Serologic burden across chronic pathogens, including herpesviruses	Elevated inflammatory tone; immune remodeling	Mortality, frailty, metabolic dysfunction, cognitive impairment	Supports a cumulative-burden model, although not all studies measured HSV, EBV, and CMV concurrently
Early-life dual herpesvirus exposure	Pediatric cohort (Generation R Study) [127]	CMV and EBV exposure (serostatus)	Effector-memory T-cell shaping without major loss of naïve responses	Long-term immune imprinting; no major vaccine impairment in young children	Persistent herpesvirus exposure may establish a durable immune tone early in life without compromising vaccine responses

**Table 2:** Herpesvirus Reactivation in Critical Illness Cohorts (including septic shock and COVID-19 ICU populations).

Study/Population	Viruses Evaluated	Key Findings	Clinical Outcomes	Reference
Septic shock cohort (previously immunocompetent adults)	Multiple herpesviruses (CMV, EBV, HSV, HHV-6)	Any herpesvirus viremia detected in 68% of patients; EBV 48%, HSV-1 26%, HHV-6 24%, CMV 18%; multiple concurrent viremia in 34%	Concurrent CMV + EBV viremia independently associated with increased mortality (adjusted SHR 3.17, 95% CI 1.41–7.13)	[83]
Severe pneumonia/mechanically ventilated patients	Multiple herpesviruses, including HSV	Viral reactivation detected in the lungs of patients with severe pneumonia	Viral reactivation associated with increased mortality	[125]
COVID-19 ICU cohort	EBV, CMV, HSV-1/2	Viral reactivation occurred in 85% of patients, with multiple herpesvirus reactivation in 63%; EBV most common (65%)	Viral reactivation independently associated with increased mortality (OR 2.46) and a higher risk of ventilator-associated pneumonia (VAP) and bloodstream infection	[84]
Critical illness cohort	CMV + HHV-6	HHV-6 viremia occurred in 23% of patients; 70% of HHV-6-positive patients also reactivated CMV	Composite endpoint of death or continued hospitalization at day 30 occurred in 76% with CMV/HHV-6 coreactivation vs. 41% without viral reactivation; adjusted OR 7.5 (95% CI 1.9–29.9)	[82]
Pooled ICU evidence	CMV, EBV, HSV ± HHV-6	Multi-herpesvirus reactivation identifies high-risk inflammatory phenotype	Associated with inflammatory dysregulation and increased mortality across multiple ICU populations	[82,83,84,125]

Note: In Lopez Roa et al. [82], the primary endpoint was a composite outcome (death or continued hospitalization at day 30) rather than mortality alone; therefore, the reported values should not be interpreted as mortality rates. Data from COVID-19 cohorts [84] are indicated separately from studies conducted in general ICU populations.

## Data Availability

All data are presented in the text.

## Abbreviations

The following abbreviations are used in this manuscript:

Akt	Protein kinase B
AP-1	Activator protein 1
APC	Antigen-presenting cell
BAFF	B-cell activating factor
BCR	B-cell receptor
CGRP	Calcitonin gene-related peptide
CMV	Cytomegalovirus
CNS	Central nervous system
COX-2	Cyclooxygenase-2
CRP	C-reactive protein
CXCL10	C-X-C motif chemokine ligand 10
EA-IgG	Early antigen immunoglobulin G (EBV)
EBNA1	Epstein–Barr nuclear antigen 1
EBV	Epstein–Barr virus
fMRI	Functional magnetic resonance imaging
HHV-6	Human herpesvirus 6
HSV	Herpes simplex virus
ICU	Intensive care unit
IFN-I	Type I interferon
IFN-γ	Interferon gamma
IgG	Immunoglobulin G
IL-6	Interleukin-6
JAK/STAT	Janus kinase/signal transducer and activator of transcription
LAT	Latency-associated transcript
LMP1	Latent membrane protein 1
LMP2A	Latent membrane protein 2A
MHC-I	Major histocompatibility complex class I
MICB	MHC class I chain-related B
MS	Multiple sclerosis
NF-κB	Nuclear factor kappa B
NKG2D	Natural killer group 2, member D
NK	Natural killer
OR	Odds ratio
PCR	Polymerase chain reaction
PET	Positron emission tomography
PGE_2_	Prostaglandin E_2_
PI3K	Phosphatidylinositol 3-kinase
PRR	Pattern-recognition receptor
SHR	Subdistribution hazard ratio
SLE	Systemic lupus erythematosus
TAC	Trigeminal autonomic cephalalgia
Tfh	T follicular helper
TLR	Toll-like receptor
TNF-α	Tumor necrosis factor alpha
TRM	Tissue-resident memory T cell
VAP	Ventilator-associated pneumonia
